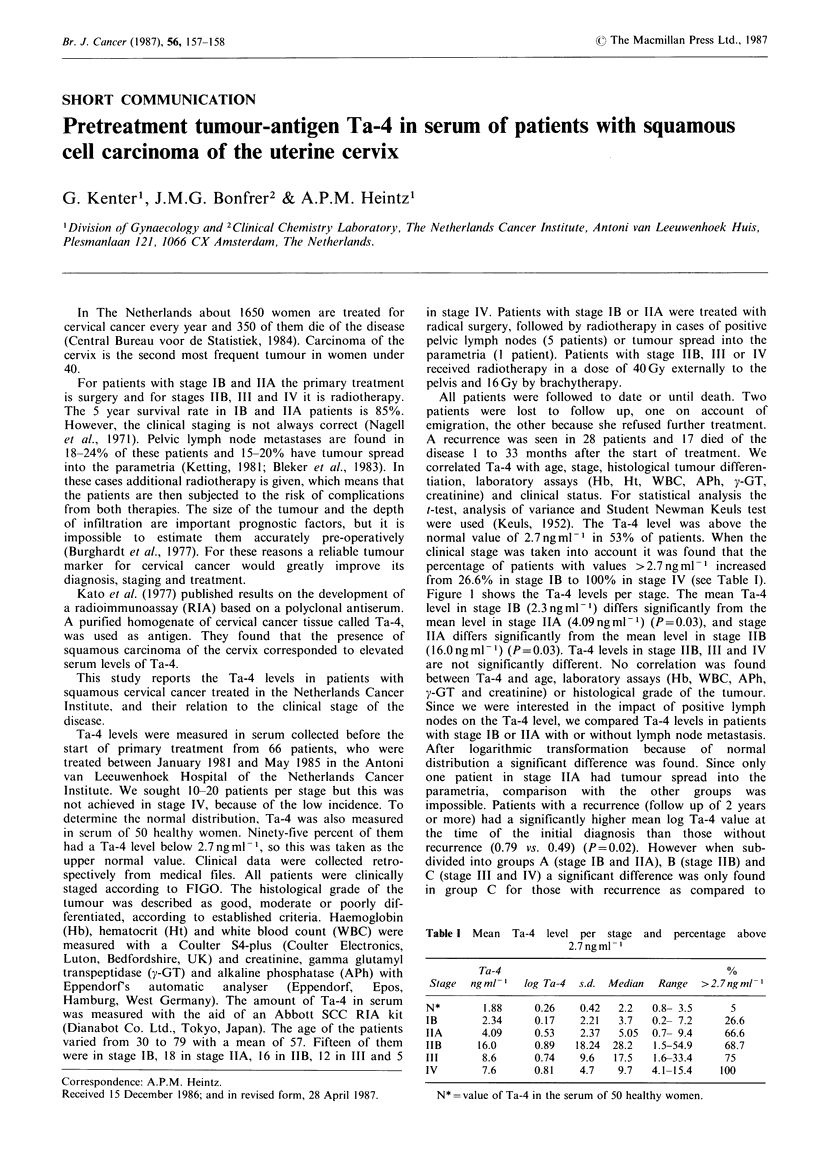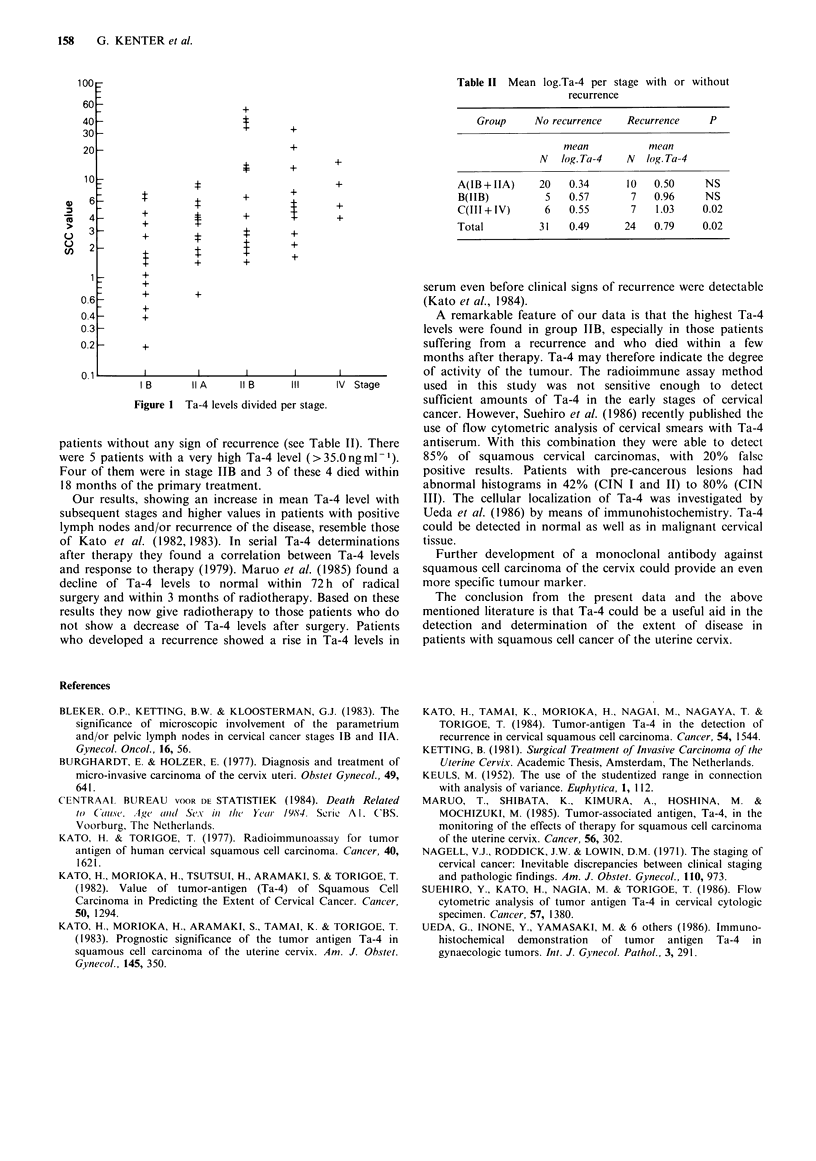# Pretreatment tumour-antigen Ta-4 in serum of patients with squamous cell carcinoma of the uterine cervix.

**DOI:** 10.1038/bjc.1987.176

**Published:** 1987-08

**Authors:** G. Kenter, J. M. Bonfrer, A. P. Heintz

**Affiliations:** Division of Gynaecology, Netherlands Cancer Institute, Amsterdam.


Br. J. Cancer (1987), 56, 157–158 © The Macmillan Press Ltd., 1987

SHORT COMMUNICATION

### Pretreatment tumour-antigen Ta-4 in serum of patients with squamous cell carcinoma of the uterine cervix

G. Kenter[1], J.M.G. Bonfrer[2] & A.P.M. Heintz[1]

[1]Division of Gynaecology and [2]Clinical Chemistry Laboratory, The Netherlands Cancer Institute, Antoni van Leeuwenhoek Huis, Plesmanlaan 121, 1066 CX Amsterdam, The Netherlands.

In The Netherlands about 1650 women are treated for cervical cancer every year and 350 of them die of the disease (Central Bureau voor de Statistiek, 1984). Carcinoma of the cervix is the second most frequent tumour in women under 40.

For patients with stage IB and IIA the primary treatment is surgery and for stages IIB, III and IV it is radiotherapy. The 5 year survival rate in IB and IIA patients is 85%. However, the clinical staging is not always correct (Nagell *et al.*, 1971). Pelvic lymph node metastases are found in 18–24% of these patients and 15–20% have tumour spread into the parametria (Ketting, 1981; Bleker *et al.*, 1983). In these cases additional radiotherapy is given, which means that the patients are then subjected to the risk of complications from both therapies. The size of the tumour and the depth of infiltration are important prognostic factors, but it is impossible to estimate them accurately pre-operatively (Burghardt *et al.*, 1977). For these reasons a reliable tumour marker for cervical cancer would greatly improve its diagnosis, staging and treatment.

Kato *et al.* (1977) published results on the development of a radioimmunoassay (RIA) based on a polyclonal antiserum. A purified homogenate of cervical cancer tissue called Ta-4, was used as antigen. They found that the presence of squamous carcinoma of the cervix corresponded to elevated serum levels of Ta-4.

This study reports the Ta-4 levels in patients with squamous cervical cancer treated in the Netherlands Cancer Institute, and their relation to the clinical stage of the disease.

Ta-4 levels were measured in serum collected before the start of primary treatment from 66 patients, who were treated between January 1981 and May 1985 in the Antoni van Leeuwenhoek Hospital of the Netherlands Cancer Institute. We sought 10–20 patients per stage but this was not achieved in stage IV, because of the low incidence. To determine the normal distribution, Ta-4 was also measured in serum of 50 healthy women. Ninety-five percent of them had a Ta-4 level below 2.7 $ng\,ml^{-1}$, so this was taken as the upper normal value. Clinical data were collected retrospectively from medical files. All patients were clinically staged according to FIGO. The histological grade of the tumour was described as good, moderate or poorly differentiated, according to established criteria. Haemoglobin (Hb), hematocrit (Ht) and white blood count (WBC) were measured with a Coulter S4-plus (Coulter Electronics, Luton, Bedfordshire, UK) and creatinine, gamma glutamyl transpeptidase ($\gamma$-GT) and alkaline phosphatase (APh) with Eppendorf's automatic analyser (Eppendorf, Epos, Hamburg, West Germany). The amount of Ta-4 in serum was measured with the aid of an Abbott SCC RIA kit (Dianabot Co. Ltd., Tokyo, Japan). The age of the patients varied from 30 to 79 with a mean of 57. Fifteen of them were in stage IB, 18 in stage IIA, 16 in IIB, 12 in III and 5 in stage IV. Patients with stage IB or IIA were treated with radical surgery, followed by radiotherapy in cases of positive pelvic lymph nodes (5 patients) or tumour spread into the parametria (1 patient). Patients with stage IIB, III or IV received radiotherapy in a dose of 40 Gy externally to the pelvis and 16 Gy by brachytherapy.

All patients were followed to date or until death. Two patients were lost to follow up, one on account of emigration, the other because she refused further treatment. A recurrence was seen in 28 patients and 17 died of the disease 1 to 33 months after the start of treatment. We correlated Ta-4 with age, stage, histological tumour differentiation, laboratory assays (Hb, Ht, WBC, APh, $\gamma$-GT, creatinine) and clinical status. For statistical analysis the $t$-test, analysis of variance and Student Newman Keuls test were used (Keuls, 1952). The Ta-4 level was above the normal value of 2.7 $ng\,ml^{-1}$ in 53% of patients. When the clinical stage was taken into account it was found that the percentage of patients with values >2.7 $ng\,ml^{-1}$ increased from 26.6% in stage IB to 100% in stage IV (see Table I). Figure 1 shows the Ta-4 levels per stage. The mean Ta-4 level in stage IB (2.3 $ng\,ml^{-1}$) differs significantly from the mean level in stage IIA (4.09 $ng\,ml^{-1}$) ($P=0.03$), and stage IIA differs significantly from the mean level in stage IIB (16.0 $ng\,ml^{-1}$) ($P=0.03$). Ta-4 levels in stage IIB, III and IV are not significantly different. No correlation was found between Ta-4 and age, laboratory assays (Hb, WBC, APh, $\gamma$-GT and creatinine) or histological grade of the tumour. Since we were interested in the impact of positive lymph nodes on the Ta-4 level, we compared Ta-4 levels in patients with stage IB or IIA with or without lymph node metastasis. After logarithmic transformation because of normal distribution a significant difference was found. Since only one patient in stage IIA had tumour spread into the parametria, comparison with the other groups was impossible. Patients with a recurrence (follow up of 2 years or more) had a significantly higher mean log Ta-4 value at the time of the initial diagnosis than those without recurrence (0.79 *vs.* 0.49) ($P=0.02$). However when subdivided into groups A (stage IB and IIA), B (stage IIB) and C (stage III and IV) a significant difference was only found in group C for those with recurrence as compared to

**Table I** Mean Ta-4 level per stage and percentage above 2.7 $ng\,ml^{-1}$

| *Stage* | *Ta-4 $ng\,ml^{-1}$* | *log Ta-4* | *s.d.* | *Median* | *Range* | *% >2.7 $ng\,ml^{-1}$* |
|---|---|---|---|---|---|---|
| N* | 1.88 | 0.26 | 0.42 | 2.2 | 0.8– 3.5 | 5 |
| IB | 2.34 | 0.17 | 2.21 | 3.7 | 0.2– 7.2 | 26.6 |
| IIA | 4.09 | 0.53 | 2.37 | 5.05 | 0.7– 9.4 | 66.6 |
| IIB | 16.0 | 0.89 | 18.24 | 28.2 | 1.5–54.9 | 68.7 |
| III | 8.6 | 0.74 | 9.6 | 17.5 | 1.6–33.4 | 75 |
| IV | 7.6 | 0.81 | 4.7 | 9.7 | 4.1–15.4 | 100 |

N* = value of Ta-4 in the serum of 50 healthy women.

Correspondence: A.P.M. Heintz.
Received 15 December 1986; and in revised form, 28 April 1987.

**Figure 1** Ta-4 levels divided per stage.

patients without any sign of recurrence (see Table II). There were 5 patients with a very high Ta-4 level ($>35.0\,ng\,ml^{-1}$). Four of them were in stage IIB and 3 of these 4 died within 18 months of the primary treatment.

Our results, showing an increase in mean Ta-4 level with subsequent stages and higher values in patients with positive lymph nodes and/or recurrence of the disease, resemble those of Kato *et al.* (1982, 1983). In serial Ta-4 determinations after therapy they found a correlation between Ta-4 levels and response to therapy (1979). Maruo *et al.* (1985) found a decline of Ta-4 levels to normal within 72 h of radical surgery and within 3 months of radiotherapy. Based on these results they now give radiotherapy to those patients who do not show a decrease of Ta-4 levels after surgery. Patients who developed a recurrence showed a rise in Ta-4 levels in

**Table II** Mean log.Ta-4 per stage with or without recurrence

| *Group* | *No recurrence* | | *Recurrence* | | *P* |
|---|---|---|---|---|---|
| | *N* | *mean log.Ta-4* | *N* | *mean log.Ta-4* | |
| A(IB+IIA) | 20 | 0.34 | 10 | 0.50 | NS |
| B(IIB) | 5 | 0.57 | 7 | 0.96 | NS |
| C(III+IV) | 6 | 0.55 | 7 | 1.03 | 0.02 |
| Total | 31 | 0.49 | 24 | 0.79 | 0.02 |

serum even before clinical signs of recurrence were detectable (Kato *et al.*, 1984).

A remarkable feature of our data is that the highest Ta-4 levels were found in group IIB, especially in those patients suffering from a recurrence and who died within a few months after therapy. Ta-4 may therefore indicate the degree of activity of the tumour. The radioimmune assay method used in this study was not sensitive enough to detect sufficient amounts of Ta-4 in the early stages of cervical cancer. However, Suehiro *et al.* (1986) recently published the use of flow cytometric analysis of cervical smears with Ta-4 antiserum. With this combination they were able to detect 85% of squamous cervical carcinomas, with 20% false positive results. Patients with pre-cancerous lesions had abnormal histograms in 42% (CIN I and II) to 80% (CIN III). The cellular localization of Ta-4 was investigated by Ueda *et al.* (1986) by means of immunohistochemistry. Ta-4 could be detected in normal as well as in malignant cervical tissue.

Further development of a monoclonal antibody against squamous cell carcinoma of the cervix could provide an even more specific tumour marker.

The conclusion from the present data and the above mentioned literature is that Ta-4 could be a useful aid in the detection and determination of the extent of disease in patients with squamous cell cancer of the uterine cervix.

### References

BLEKER, O.P., KETTING, B.W. & KLOOSTERMAN, G.J. (1983). The significance of microscopic involvement of the parametrium and/or pelvic lymph nodes in cervical cancer stages IB and IIA. *Gynecol. Oncol.*, **16**, 56.

BURGHARDT, E. & HOLZER, E. (1977). Diagnosis and treatment of micro-invasive carcinoma of the cervix uteri. *Obstet Gynecol.*, **49**, 641.

CENTRAAL BUREAU VOOR DE STATISTIEK (1984). *Death Related to Cause, Age and Sex in the Year 1984.* Serie A1, CBS, Voorburg, The Netherlands.

KATO, H. & TORIGOE, T. (1977). Radioimmunoassay for tumor antigen of human cervical squamous cell carcinoma. *Cancer*, **40**, 1621.

KATO, H., MORIOKA, H., TSUTSUI, H., ARAMAKI, S. & TORIGOE, T. (1982). Value of tumor-antigen (Ta-4) of Squamous Cell Carcinoma in Predicting the Extent of Cervical Cancer. *Cancer*, **50**, 1294.

KATO, H., MORIOKA, H., ARAMAKI, S., TAMAI, K. & TORIGOE, T. (1983). Prognostic significance of the tumor antigen Ta-4 in squamous cell carcinoma of the uterine cervix. *Am. J. Obstet. Gynecol.*, **145**, 350.

KATO, H., TAMAI, K., MORIOKA, H., NAGAI, M., NAGAYA, T. & TORIGOE, T. (1984). Tumor-antigen Ta-4 in the detection of recurrence in cervical squamous cell carcinoma. *Cancer*, **54**, 1544.

KETTING, B. (1981). *Surgical Treatment of Invasive Carcinoma of the Uterine Cervix.* Academic Thesis, Amsterdam, The Netherlands.

KEULS, M. (1952). The use of the studentized range in connection with analysis of variance. *Euphytica*, **1**, 112.

MARUO, T., SHIBATA, K., KIMURA, A., HOSHINA, M. & MOCHIZUKI, M. (1985). Tumor-associated antigen, Ta-4, in the monitoring of the effects of therapy for squamous cell carcinoma of the uterine cervix. *Cancer*, **56**, 302.

NAGELL, V.J., RODDICK, J.W. & LOWIN, D.M. (1971). The staging of cervical cancer: Inevitable discrepancies between clinical staging and pathologic findings. *Am. J. Obstet. Gynecol.*, **110**, 973.

SUEHIRO, Y., KATO, H., NAGIA, M. & TORIGOE, T. (1986). Flow cytometric analysis of tumor antigen Ta-4 in cervical cytologic specimen. *Cancer*, **57**, 1380.

UEDA, G., INONE, Y., YAMASAKI, M. & 6 others (1986). Immunohistochemical demonstration of tumor antigen Ta-4 in gynaecologic tumors. *Int. J. Gynecol. Pathol.*, **3**, 291.